# 3D Digital Image Correlation Analysis of the Shrinkage Strain in Four Dual Cure Composite Cements

**DOI:** 10.1155/2019/2041348

**Published:** 2019-11-17

**Authors:** Aleksandra Mitrović, Dušan Antonović, Ivan Tanasić, Nenad Mitrović, Gordana Bakić, Dejana Popović, Miloš Milošević

**Affiliations:** ^1^University of Belgrade, Faculty of Technology and Metallurgy, Karnegijeva 4, 11000 Belgrade, Serbia; ^2^University of Belgrade, School of Dental Medicine, Rankeova 4, 11000 Belgrade, Serbia; ^3^University of Belgrade, Faculty of Mechanical Engineering, Kraljice Marije 16, 11000 Belgrade, Serbia; ^4^University of Belgrade, Institute of Nuclear Sciences Vinca, 12-14 Mike Petrovića Street, 11000 Belgrade, Serbia; ^5^Innovation Center of Faculty of Mechanical Engineering in Belgrade, Kraljice Marije 16, 11000 Belgrade, Serbia

## Abstract

The introduction of resin-based cements and an adhesive-bonding system in daily dental practice has given the opportunity to increase the retention of previously conventional cemented restorations and the optimal results in esthetic. This experimental study employed the 3D Digital Image Correlation Method (3D-DIC) for detecting shrinkage strain in four dual cured composite cements. The aim was to visualize measure, analyze, and compare strain fields in four resin-based cements using the 3D-DIC method. A total of 72 samples were divided into 4 groups considering variations in sample types, diameter, and thickness. Four types of composite cements: RelyX U200 (3 M ESPE, St. Paul, MN, USA), MaxCem Elite (Kerr, Orange, CA, USA), Multilink Automix (Ivoclar Vivadent, Schaan, Liechtenstein), and SeT PP (SDI, Australia) were used. Each type had diameters of 3 mm, 4 mm, and 5 mm, respectively, combined with two different values of thickness: 1 mm and 2 mm. Thickness had an important role on strain detected in all tested materials showing higher strain in samples with 2 mm thickness compared to 1 mm samples. Shrinkage strain values were the highest in Set PP samples indicated the possibility of undesirable de-bonding.

## 1. Introduction

In current dental practice resin-based cements (RBCs) have usually been used for all ceramic restorations fixations, since they overcome poor mechanical, biological, and adhesion features of the previously used cement [1, 2]. The right choice of RBCs is significant for the longevity of dental restorative materials. Composition of each ceramic system type is unique and therefore requires appropriate type of resin-based cements and cementation protocol. Following the protocol, it is important to establish adequate bond between cement and ceramic agents through adhesive or self-adhesive bonding [3–5].

Mechanical properties of the RBCs used for the cementation of all ceramics could determine long-term clinical prognosis due to adhesion problems. Improved therapy success using resin composite cements is primarily based on their significant properties [6], such as thermal and chemical stability, decreased hydrolytic degradation, better solubility, wear resistance, higher elasticity, plasticity, hardness, and strength.

The standard protocol steps prior to cementation, such as conditioning or priming pretreatments of tooth, involve using composite cements due to acidic and hydrophilic monomers and their ability to create stable chemical composition responsible for the strong bonding [7]. These monomers are also responsible for polymerization shrinkage stress and shrinkage strain, manifested during the RBCs' hardening process [8, 9]. Additionally, curing mode and filler content have also been found to be important for strain magnitude [9].

Dual cure mode, consisting of self-cure and light cure mode, has demonstrated the mechanical properties' enhancement. The polymerization shrinkage strain in RBCs during the hardening process can cause therapeutic failure due to de-bonding [10]. Namely, strain detected when converting monomer to polymer leads to micro-leakage and has adverse effect on longevity of restorations due to cement degradation [11–13]. In addition, the thickness of the cement layer has been found to be an important factor for the cementation [14].

We have conducted an experimental study using the 3D Digital Image Correlation Method (3D-DIC) as an optical technique for detecting shrinkage strain in composite cements immediately after photo-polymerization. The main goal of the study was to determine, analyze, and compare strain fields in four RBCs using the 3D-DIC method, since the shrinkage of RBCs may compromise its bonding effectiveness.

The following hypotheses of study were formulated:

(a) Strain values differ in all four tested cements.
(b) Sample thickness influences strain values.
(c) Sample diameter influences strain values.

## 2. Materials and Methods

Strain field was measured using the 3D optical system Aramis 2 M (GOM, Braunschweig, Germany) based on 3D-DIC method [15]. Prior to the experiment, system calibration was performed using the calibration panel for corresponding measurement volume. The volume was chosen based on the dimensions of the measured area on the sample surface. After successful calibration, the measuring could begin.

A total of 72 samples were divided into 4 groups. The groups included 4 types of composite cements: RelyX U200 (3 M ESPE, St. Paul, MN, USA), MaxCem Elite (Kerr, Orange, CA, USA), Multilink Automix (Ivoclar Vivadent, Schaan, Liechtenstein) and SeT PP (SDI, Australia). Each type had diameters of 3 mm, 4 mm and 5 mm, combined with two different values of thickness: 1 mm and 2 mm. Samples were prepared by filling ring-type molds. The top surface of each sample was sprayed with fine black and white spray (Kenda Color Acrilico, Kenda Farben) to create a stochastic pattern with high contrast for image analysis. Digital images were made immediately prior and after light curing. A LED light-curing unit (450–500 mW/cm^2^, LEDition, Ivoclar-Vivadent, Schaan, Liechtenstein) was used for 40 s to activate polymerization. The images were then analyzed using special software (Aramis 6.2.0) to determine the von Mises strain. Analysis of the von Misses strain fields was done using three sections (Sections 0, 1, and 2). Section 0 is a circular section positioned on the material-mold interface of the sample. Sections 1 and 2 are linear sections positioned orthogonally to each other. Diameter of the Section 0 and length of the Sections 1 and 2 correspond to sample diameter.

All tested materials are dual cure. MaxCem Elite and SeT PP are self-adhesive and self-etch cements. Light-curing mode was used. The experiments were done at room temperature.

The obtained data were statistically analyzed using the general linear model (GLM) for factors “material”, “diameter” and “thickness”, as well as their interactions. When the interaction was significant (*p* < 0.05), one-way analysis of variance (ANOVA) was performed with Tukey's post-hoc tests and Bonferroni correction. The level of significance was set at *p* = 0.05. Statistical analysis was performed using the software package Minitab 16 (Minitab Inc., State College, PA, USA).

## 3. Results

**Figure 1:**
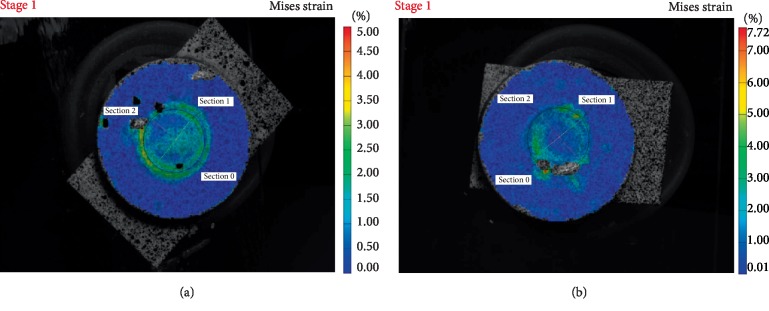
Von Mises strain field of MaxCem Elite across the sample surface showing the outer segment with higher and inner segments with lower strain values (a) sample photograph with overlaying Von Mises strain field for1 mm thickness; (b) sample photograph with overlaying Von Mises strain field for 2 mm thickness.

**Figure 2:**
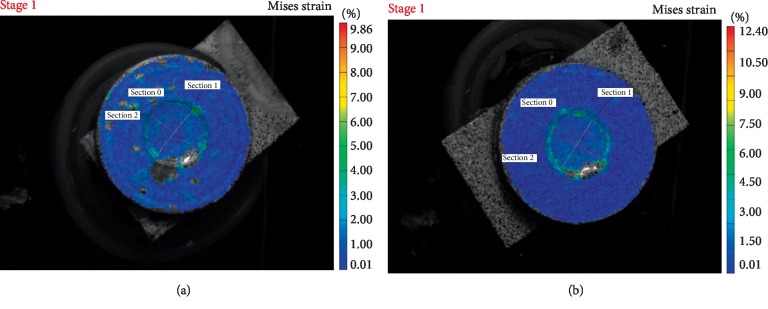
Von Mises strain field of SeT PP across the sample surface showing the outer segment with higher and inner segments with lower strain values (a) sample photograph with overlaying Von Mises strain field for1 mm thickness; (b) sample photograph with overlaying Von Mises strain field for 2 mm thickness.

**Figure 3:**
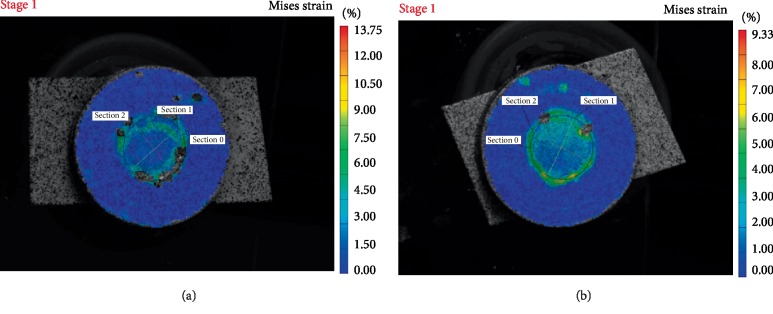
Von Mises strain field of Relyx U200 across the sample surface showing the outer segment with higher and inner segments with lower strain values (a) sample photograph with overlaying Von Mises strain field for1 mm thickness; (b) sample photograph with overlaying Von Mises strain field for 2 mm thickness.

**Figure 4:**
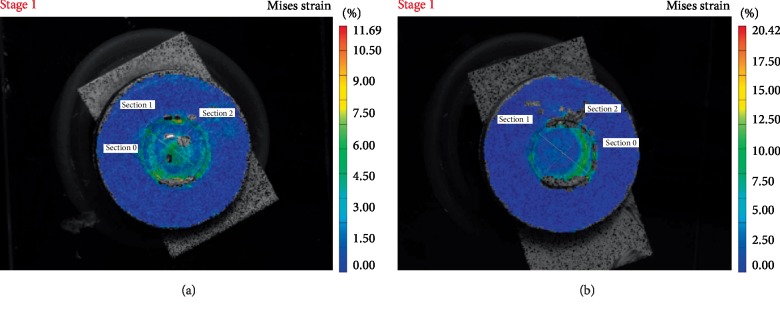
Von Mises strain field of Multilink Automix across the sample surface showing the outer segment with higher and inner segments with lower strain values (a) sample photograph with overlaying Von Mises strain field for1 mm thickness; (b) sample photograph with overlaying Von Mises strain field for 2 mm thickness.

Figures 1–4 are representative images of the von Mises strain across the surface of each material with diameter 5 mm after photo-polymerization. Maximum von Misses strain values were measured in Section 0 in all figures. As seen in Figures 1–4, all composite cements sized Ø5 × 2 mm expressed higher strain values in Section 0 compared to Ø5 × 1 mm samples. Furthermore, MaxCem Elite and Multilink Automix samples Ø5 × 1 mm showed a similar distribution of the highest strain values in all Sections. However, Relyx U200 and SeT PP samples Ø5 × 2 mm showed similar distribution of the highest strain values in all Sections.

Strain mean revealed that the von Misses strain in Section 0, Section 1 and Section 2 did not depend on the diameter of the samples (Table 1). Samples with 2 mm thickness indicated significantly higher values of the von Misses strain in all Sections compared to the samples with 1 mm thickness. The von Mises strain was significantly higher in Section 0 than in Sections 1 and 2 for all composite cements. A significant difference regarding the thickness of the material and the type of composite cement (*p* = 0.002) has been found (Table 2). A significant difference (*p* = 0.001) in strain values has been found between the type of cement, thickness and diameter in Sections 1 and 2 (Table 2). Furthermore, ANOVA showed that with 1 mm thickness, Multilink Automix exhibited higher von Misses strain values compared to others. Considering samples with 1 mm thickness, Multilink Automix and MaxCem Elite showed significantly higher strain in Section 1 and Section 2 compared to RelyX U200 and SeT PP. RelyX U200 showed higher von Misses strain values with 2 mm thickness. Significantly higher values of von Misses strain were measured for Multilink Automix and RelyX U200 compared to SeT PP and MaxCem Elite in all Sections with 2 mm thickness.

## 4. Discussion

The study aimed to determine strain immediately after the polymerization of four composite cements in the ring type molds. It is important to emphasize that the quality of the specimen can influence the outcome of the test, as the RBCs were molded into the required test shape and then polymerized. It should be ensured that the material is adequately and uniformly cured for the appropriate amount of time and with sufficient light energy. These conditions provide the most valid test results and characterize the given material's optimal attainable properties [3].

The results presented in this study indicated nonuniform strain distribution in all tested materials. The nonuniform distribution of shrinkage strain was also reported using a single digital image/camera 2D digital image correlation, showing the highest shrinkage in subsurface parts of the sample [16]. Similarity has been found in the work of Oliveira et al. [17] who noticed increased polymerization shrinkage at the adhesive interface and possible adhesive failure. Given that intermolecular distance between the monomers is replaced by a covalent bond, polymerization shrinkage and resulting shrinkage strain are inherent to polymerization. Differences in chemical composition and filler type or size have directly influenced the shrinkage behavior. The monomer-chain mobility can be limited by the amount of the fillers, leading to decreased monomers and radical mobility, so resulting in lower shrinkage [13, 18]. However, manufacturers do not state the precise chemical composition of all tested materials, so it is difficult to compare strain in this respect.

Previous studies [6, 10, 19] have been conducted on the standardized sample size showing data on strain mean. Our study cannot be compared to others, as they focused on the overall volumetric shrinkage, rather than deformation fields. Using two cameras system, 3D-DIC technique provided sufficient data about out-of-plane shrinkage strain [15]. An advantage of the 3D-DIC method over other methods is the ability of full-field strain measurement [9, 20–23]. These methods excluded peripheral section (Section 0) although the peripheral strain has to be considered when interpreting the overall strain. 3D-DIC method additionally detected maximal von Misses strain values and determined the zones of the maximal strain through presenting images of 3D full strain field. Including several sizes of experimental samples, our study revealed additional information about sample size dependent shrinkage patterns focusing on strain field.

Increased, nonuniformly distributed shrinkage strain at adhesive interface induced by shrinkage of RBCs may compromise bonding and lead to adhesive failure. This may be of particular importance in the case of ceramic systems. Shrinkage strain values were the highest in SeT PP samples, which indicated the possibility of undesirable de-bonding. Also, finding the optimal thickness of RBC film could prevent fracture of ceramics [24–26]. De-bonding of all ceramic systems is correlated with cement thickness as a consequence of adhesion failure due to occlusal loads [27]. High shrinkage values of RBCs, as seen in the present study, may compromise their bonding effectiveness.

## 5. Conclusion

The study investigated samples of four different dual cure composite cements used in a daily dental practice, using a modern noncontact method—the 3D Digital Image Correlation. Within the limits of 3D-DIC method and the selection of tested materials, we can conclude the following: statistics showed that the von Mises strain in the peripheral zone of samples (Section 0) did not depend on the diameter of the samples, although correlation was found between thickness and peripheral zones of the samples. The two out of three hypotheses of study were confirmed. Thickness had an important impact on strain detected in all four composite cements, showing higher strain in samples with 2 mm thickness compared to 1 mm samples. The advantage of the 3D-DIC method is the ability to measure the highest strain values and to visualize the highest strain locations in tested samples. In addition, shrinkage strain values were the highest in SeT PP samples, indicating the possibility of undesirable de-bonding. Further investigation should include the role of each RBC component on the final properties of the materials and its bonding to the ceramics.

## Figures and Tables

**Table 1:** Strain measurements of four different composite cements with variations in size and thickness.

Material	Thickness	Diameter	Mean strain, section 0 von Misses, %	Standard deviation for section 0	Mean strain, section 1 von Misses, %	Standard deviation for section 1	Mean strain, section 2 von Misses, %	Standard deviation for section 2	Highest strain value section 0 von Misses, %
Maxcem Elite Kerr	1	3	2.1733	0.2528	1.7794	0.0167	1.8207	0.3287	6.65
		4	2.3324	0.1362	2.1653	0.5626	1.8980	0.6922	7.14
		5	1.9741	0.6387	1.8365	0.5083	1.6301	0.5977	5.65
	2	3	1.7405	0.3595	1.2874	0.4056	1.3886	0.4950	5.01
		4	2.3064	0.3152	1.4679	0.0712	1.4825	0.1738	6.33
		5	2.2836	0.3043	0.9359	0.3291	0.8568	0.0832	5.49
Multilink Automix Ivoclar Vivadent	1	3	2.2811	0.6781	1.6517	0.9899	2.1128	0.9083	6.10
		4	2.5541	0.3745	1.9505	0.8885	1.9443	0.5436	6.22
		5	3.3695	0.4611	2.8395	0.2977	3.0017	0.3475	9.74
	2	3	2.9937	0.2701	2.9829	1.8836	2.8327	1.3025	9.58
		4	2.7502	0.4123	1.8787	0.3942	1.6329	0.2572	5.94
		5	4.0263	1.4931	2.4652	0.5226	1.9701	0.7203	10.41
Relyx U200	1	3	2.3289	0.4769	1.6830	1.1743	1.1334	0.4411	4.41
		4	2.4721	1.2366	1.3668	1.0619	1.1931	0.9892	7.50
		5	2.2387	0.9129	0.7980	0.2231	0.8445	0.3176	10.42
	2	3	4.0793	0.4662	1.4816	0.5275	1.6820	0.6401	9.24
		4	4.6121	0.6277	1.6257	0.3809	1.6915	0.2917	8.33
		5	3.6284	0.3406	2.1172	0.2955	2.1496	0.3538	10.03
Set PP SDI	1	3	2.1314	0.7654	0.7795	0.0738	0.9144	0.1064	11.16
		4	1.6187	0.3665	1.0102	0.3971	0.8125	0.1432	9.29
		5	1.7964	0.0922	1.1519	0.6241	0.9696	0.4441	7.21
	2	3	2.2818	1.2817	1.8395	0.5557	2.1128	1.1096	6.71
		4	3.2813	0.7691	1.8935	0.5955	2.0672	0.9814	7.26
		5	2.0264	0.8074	0.9483	0.2069	0.9383	0.1031	5.61

**Table 2:** Analysis of variance for von mises, using adjusted SS for tests.

Source	The total degrees of freedom (DF)		The sequential sums of squares (Seq SS)		Adjusted sums of squares (Adj SS)		F‐value		P‐value	
	Section 0	Section 1+ 2	Section 0	Section 1+ 2	Section 0	Section 1+ 2	Section 0	Section 1+ 2	Section 0	Section 1+ 2
Material	3	3	16.7169	7.3716	16.7169	7.3716	5.5723	20.93	0.001	0.001
Thickness (mm)	1	1	9.5469	0.8899	9.5469	0.8899	9.5469	7.58	0.001	0.007
Diameter (mm)	2	2	0.7245	0.2412	0.7245	0.2412	0.3622	1.03	0.460	0.361
Correlation between material, thickness and diameter	6	6	2.5884	2.8495	2.5884	2.8495	0.4314	4.04	0.476	0.001∗∗
Correlation between material and thickness	3	3	7.7163	5.5469	7.7163	5.5469	2.5721	15.75	0.002∗	0.001
Correlation between material and diameter	6	6	6.0954	1.7609	6.0954	1.7609	1.0159	2.50	0.058	0.026
Correlation between thickness and diameter	2	2	0.6624	0.4927	0.6624	0.4927	0.3312	2.10	0.492	0.127
Error	48	120	22.0597	14.0900	22.0597		0.4596			
Total	71	143	66.1105	33.2427						

^*^Statistically significant correlation between cement type and diameter for the section 0.

^**^significant correlation between material thickness and diameter regarding sections 1 and 2.

## Data Availability

The von Mises results and statistical data used to support the findings of this study are included within the article. Also, the von Mises results and statistical data used to support the findings of this study are available from the corresponding author upon request.
